# Wearable Leg Movement Monitoring System for High-Precision Real-Time Metabolic Energy Estimation and Motion Recognition

**DOI:** 10.34133/research.0214

**Published:** 2023-08-23

**Authors:** Jinfeng Yuan, Yuzhong Zhang, Shiqiang Liu, Rong Zhu

**Affiliations:** State Key Laboratory of Precision Measurement Technology and Instruments, Department of Precision Instrument, Tsinghua University, Beijing 100084, China.

## Abstract

Comprehensive and quantitative assessment of human physical activity in daily life is valuable for healthcare, especially for those who suffer from obesity and neurological disorders or are at high risk of dementia. Common wearable devices, e.g., smartwatches, are insufficient and inaccurate for monitoring highly dynamic limb movements and assessing human motion. Here, we report a new wearable leg movement monitoring system incorporating a custom-made motion sensor with machine learning algorithm to perceive human motion accurately and comprehensively during diverse walking and running actions. The system enables real-time multimodal perceptions of personal identity, motion state, locomotion speed, and energy expenditure for wearers. A general law of extracting real-time metabolic energy from leg movements is verified although individual gaits show differences. In addition, we propose a novel sensing configuration combining unilateral lower leg movement velocity with its angular rate to achieve high accuracy and good generalizability while simplifying the wearable system. Advanced performances in personal identification (accuracy of 98.7%) and motion-state recognition (accuracy of 93.7%) are demonstrated. The wearable system also exhibites high-precision real-time estimations of locomotion speed (error of 3.04% to 9.68%) and metabolic energy (error of 4.18% to 14.71%) for new subjects across various time-varying conditions. The wearable system allows reliable leg movement monitoring and quantitative assessment of bodily kinematic and kinetic behaviors during daily activities, as well as safe identity authentication by gait parameters, which would greatly facilitate smart life, personal healthcare, and rehabilitation training.

## Introduction

Physical activity refers to the bodily movement produced by skeletal muscles that require energy expenditure. Physical activity is closely related to human health, has been shown to affect mental health [1,2] and body mass index [3–5], and can be linked to various diseases [6,7]. Long-term physical sedentary lifestyle and inactivity have become one of the main causes of obesity [8], cardiovascular diseases [9,10], and premature death [11,12]. Daily activity plays an important role in people’s health and physical rehabilitation of patients [13]. Because of this, many guidelines and policies have been implemented to promote human physical activity [14]. Human motion analysis involves multimodal information oriented to various applications, e.g., human gait pattern tied to specific personal biometric identity [15], recognition of motion state and gait to analyze motor function [16–18], real-time estimations of locomotion speed [19], and metabolic energy expenditure [20,21] to facilitate healthy and adaptive physical training.

Biometric technologies using fingerprints and facial features have been widely used in personal identifications. Because of easy replicability, these technologies remain security problems [22,23]. In comparison, personal identification based on human gait [16,24] is one of the safest and the most reliable biometric recognition modalities [25,26] because individual gait characteristics are unique. It is almost impossible for a person to imitate or counterfeit the gait of others [27]. Common methods of gait recognition utilize computer vision [28], but the recognition accuracy is affected by clothing and environmental conditions. Motion-state recognition [29–31] helps to understand the temporal characteristics of various kinds of activities, so as to make better activity configurations and build a better activity-specific model [32–34]. In our daily life, typical motion states are ambulation-type activities, such as level walking, level running, loaded walking, slope walking, and slope running, which account for the main proportion of physical activities [35]. Monitoring approaches using an accelerometer, magnetometer, gyroscope, or electromyography comprise the majority of methods to discern motion states [36–39]. However, these devices suffer from serious drift and instability problems in capturing the long-term dynamic motions of limbs [40]. Locomotion speed [41] and metabolic energy expenditure [42,43] are key metrics to objectively characterize body kinematics and kinetics in human physical activity. The measurement of locomotion speed usually depends on Global Positioning System positioning that has significant errors in areas with obstacles and is unsuitable for tracking indoor activities [44]. Using self-contained devices to produce speed estimations independent of external locators is crucial for personal daily use. As for metabolic energy measurements, clinical respirometry [45–47] is a gold standard equipment but is not feasible for personal daily use due to its expensive and intrusive breath-based measurement [48,49]. Clearly, it would be useful to have a wearable activity and metabolism monitoring system with a simple, low-cost, and wearable setup. Step counts using pedometers, smartphones, or smartwatches are common ways to measure activity [50–52]; however, there are significant deviations between step counts and ground-truth motion energy. Existing wearable devices fail to monitor leg movement that accounts for the vast majority of bodily activities and contributes the most to energy expenditure. Using inertial measurement units (IMUs) [53,54] worn on the legs may be a solution. However, the metabolic estimation error of a wearable system using 2 IMUs worn on the shank and thigh is still larger than 13% during steady-state conditions and 23% during time-varying conditions [20]. In addition, a lot of research works are devoted to monitoring human hand movements, and such systems are widely used in human–computer collaboration [55,56], sign language recognition [57], and virtual reality [34]. Up to now, wearable, miniaturized, point-of-care testing devices for comprehensively understanding human physical activity are of great significance but have not yet been thoroughly investigated.

Here, we present a new wearable leg movement monitoring system that can accurately and reliably monitor leg movements and allow a comprehensive analysis of human motion during various walking and running conditions. The wearable system worn on a lower leg (below the knee) is composed of a custom-made micro-tri-axis velocity sensor and a micro-IMU and is capable of detecting tri-axis motion velocities, accelerations, angular rates, and attitude angles of the lower leg. The leg motion parameters are fused using a machine learning algorithm to implement real-time multimodal perceptions, including recognizing personal identity and motion states, estimates of locomotion speed, and metabolic energy expenditure during various walking and running scenarios. To overcome drift and instability problems in capturing long-term dynamic motion of lower leg [58], for the first time, we propose an optimum sensing configuration for versatile tasks and prove that the combination of single shank movement velocity and angular rate is the best sensing configuration for achieving high accuracy while simplifying the wearable system. In addition, we verify individual gaits exhibit significant differences that are used to implement personal identification and motion-state recognition. Besides, we propose and verify an interesting indication that the kinetic-energy-related locomotion speed and metabolic rate can be extracted from the leg movements regardless of individual gait differences. The proposed wearable system has good generalizability that can accurately estimate the real-time locomotion speeds and metabolic energy expenditures for new subjects across various motion conditions without relying on subject-specific calibration, which is essential for practical application and large-scale deployment.

## Results and Discussion

### Overview of the wearable system

**Fig. 1.**
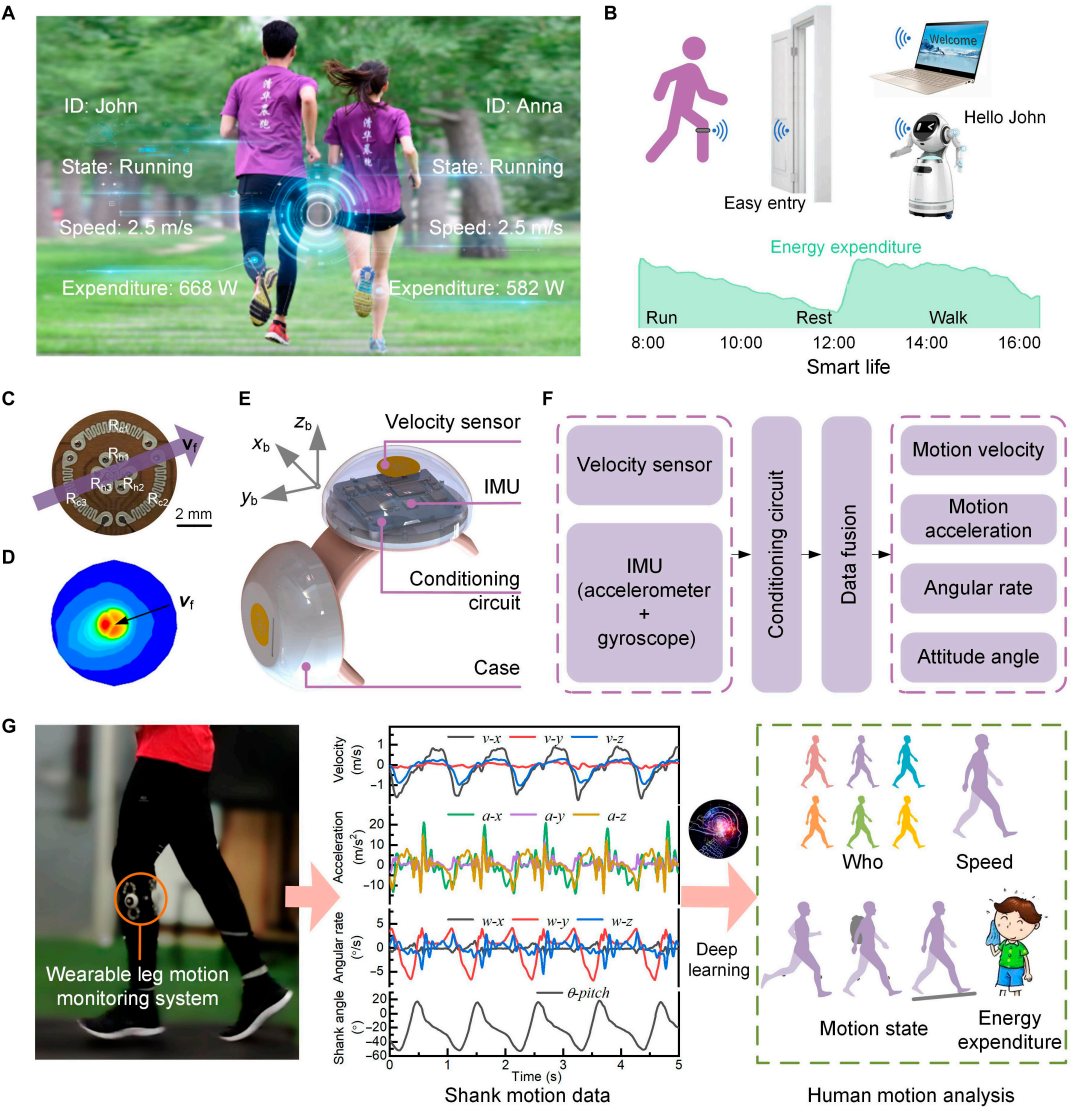
The wearable leg motion monitoring system and its multimodal functions. (A) The wearable system monitored and analyzed human motion in real time. The multimodal functions of the wearable system included identity recognition, motion state identification, and estimations of locomotion speed and metabolic energy expenditure. (B) The wearable system could enable a “smart” life with automatic identity authentication for safe and easy access to living facilities and understanding daily activity for healthy exercise management. (C) View of a microflow sensor. (D) Deflection of the thermal field caused by motion-induced surface flow. (E) Design and configuration of the wearable device. (F) Signal flow of the wearable device for deducing tri-axis motion velocity, acceleration, angular rate, and attitude angle. (G) The wearable system was worn on the shank of one leg. The detected shank movement data were fused to recognize the personal identity and motion states and estimate the locomotion speed and metabolic energy expenditure in real time by a deep learning neural network.

As a unique human biometric identity, lower limb movement reflects the individual personal manner of walking and running. We develop a wearable leg monitoring system to detect lower limb movements. Through a motion data fusion based on deep learning, the wearable system can report personal information in real time during walking and running as shown in Fig. 1A, e.g., “John is running with a locomotion speed of 2.5 m/s and a metabolic energy expenditure of 668 W. Anna is running at the same speed with a metabolic expenditure of 582 W”. With this wearable system, people can partake in a “smart” life [59,60], benefiting from safe and easy identity authentication in accessing living facilities and quantitatively assessing daily activity for healthy exercise management (Fig. 1B). A real-time demonstration is shown in Movie S1.

Here, the lower limb movement is detected using our homemade wearable device, which is composed of a custom microvelocity sensor and a commercial micro-IMU (LSM9DS1, STMicroelectronics). The microvelocity sensor is made up of 2 microflow sensors placed orthogonally to measure tri-axis motion velocity by detecting the motion-induced surface flow vectors. As shown in Fig. 1C, the microflow sensor consists of 3 center platinum resistors (R_h1_, R_h2_, and R_h3_) that are electrically heated and work as the temperature sensors of themselves for detecting the motion-induced surface flow based on convective heat transfer, while the circular platinum resistors (R_c1_, R_c2_, and R_c3_) work as the ambient temperature sensors. The thin-film platinum resistors (100 nm in thickness) are deposited and patterned on a polymide substrate by a lift-off micromachining process. The combination of 3 thermistors is used to detect simultaneously the magnitude and the 2-dimensional direction of motion velocity (*V*_f_) (Fig. 1D). By combining 2 flow sensors placed orthogonally (shown in Fig. 1E), tri-axis motion velocity can be figured out by making data fusion of 2 flow sensors. To accurately measure the tri-axis velocity of leg movement in real time during walking and running, we propose a long short-term memory (LSTM) neural network model to make data fusion and predict the dynamic motion velocity. The detailed design and testing results of the velocity measurement are described in Note S1 and Fig. S1. The results evidence the tri-axis motion velocity of the limb can be accurately measured in real time. The commercial IMU is used to detect tri-axis acceleration and tri-axis angular rate. The motion acceleration and attitude angles of the limb are figured out by a Kalman filter [58]. The microvelocity sensor and the IMU are sampled and filtered by an integrated microcontrol unit and then transmitted wirelessly to an external terminal through Bluetooth for making data fusion to figure out tri-axis motion velocity, acceleration, angular rate, and attitude angle in real time. Therefore, the wearable device worn on the shank capably detects tri-axis motion velocity (*v*), acceleration (*a*), angular rate (*w*), and attitude angle (*θ*) of the shank during walking and running (Fig. 1F). The detected shank motion parameters are configured and fused to recognize personal identity traits and motion state and estimate locomotion speed and metabolic energy expenditure in real time by a data-driven model based on deep learning (Fig. 1G).

In terms of motion data acquisition, we conducted a series of experiments and collected the motion dataset of 16 subjects (subjects 1 to 16) during their walking and running on a level treadmill (called level walking and level running), 6 subjects (subjects 1 to 6) during walking on a level treadmill and carrying a backpack of 12 kg (called loaded walking), and 6 subjects (subjects 1 to 6) during walking and running on a sloped treadmill (called slope walking and slope running). All experiments were performed twice. The subjects removed and rewore the monitoring device between 2 experiments to validate the feasibility and generalization across different wearing.

### Recognition of personal identity and motion state

**Fig. 2.**
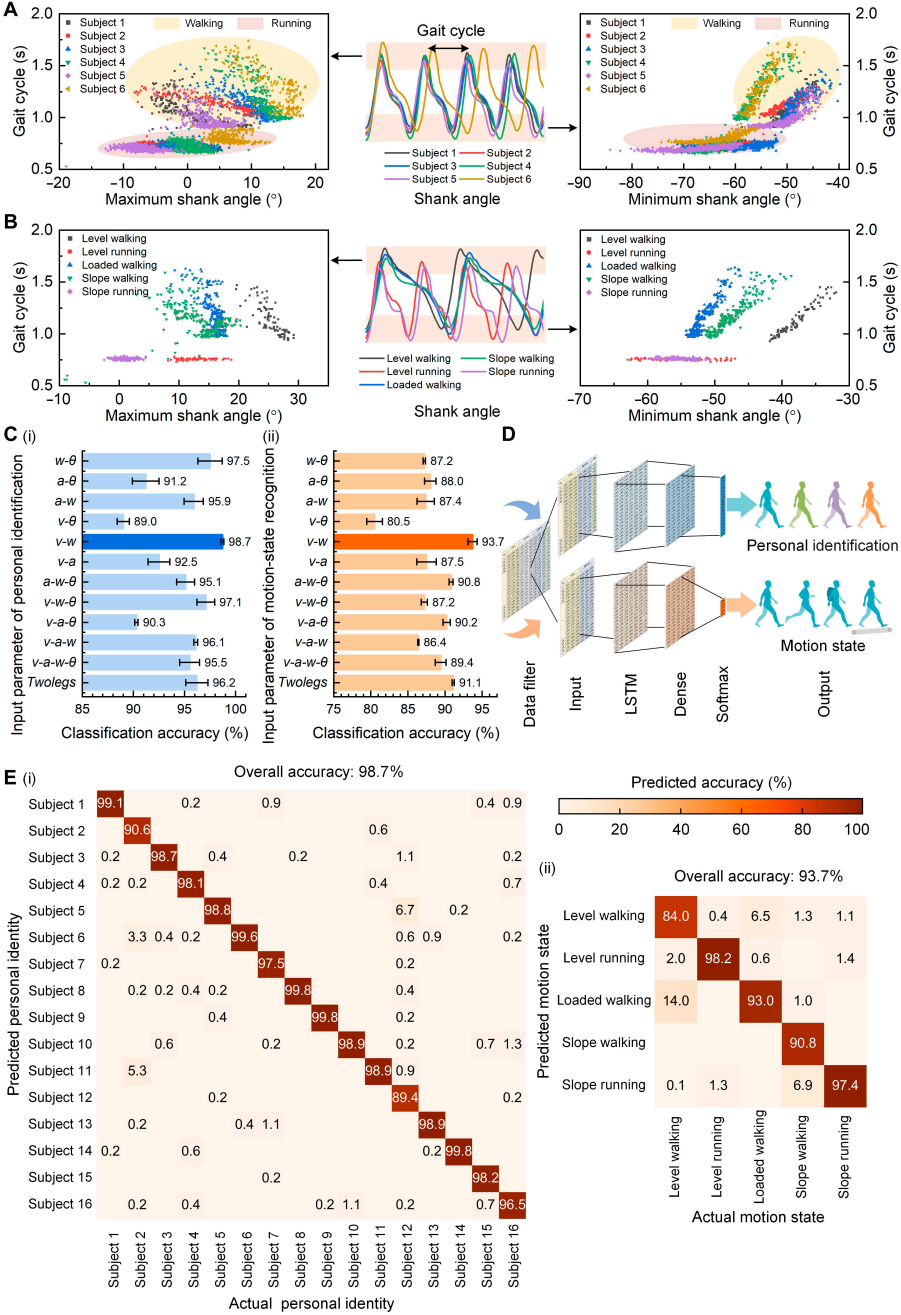
Recognition of personal identity and motion state by monitored shank movements. (A) Characteristic scatter diagrams of the gait cycles of 6 subjects. Gait characteristics during walking and running showed significant differences across subjects. (B) Characteristic scatter diagrams of the gait cycles of one subject during various motion states. The gait characteristics of one subject showed significant differences across various motion states. (C) (i) Recognition accuracies of personal identity using different input parameter configurations. (ii) Recognition accuracies of motion state using different input parameter configurations. In (i) and (ii), the best configuration with the highest accuracy was highlighted using a darker color. (D) Schematic diagram of the LSTM neural network model used for personal identification and motion-state recognition. (E) (i) Recognition test confusion matrix with 7,500 groups of the test dataset for recognizing 16 subjects. (ii) Recognition test confusion matrix with 6,000 groups of the test dataset for identifying 5 types of motion states; each row and column represented an instance in an actual category and a predicted category, respectively, and the diagonal values represented correct percentage results for each category. The color bar represented the predicted accuracy.

As mentioned above, the human gait pattern of leg movements is unique and hard to replicate and is potentially a safer biometric identifier. Figure 2A shows the characteristic scatter diagrams of the gait cycles of 6 subjects (subjects 1 to 6) during their walking and running. We selected the maximum/minimum shank angle and gait cycle length as the gait features to show the significant differences among individuals. It was seen that each person had a unique gait pattern that could be a biometric identifier. We also observed the gait pattern varied with the motion states as shown in Fig. 2B. Through analyzing the gait pattern of leg movements, the motion states could be also identified.

To perform personal identification, we built an LSTM neural network as a recognition model, considering its capability of learning long-term dependencies for sequence modeling [61,62]. The input parameter configuration of the recognition model was crucial and needed to be optimized according to both the accuracy and simplicity of the wearable system. We compared different input configurations that used: all motion parameters of bilateral shanks on both legs (*TwoLegs*), all motion parameters of a single unilateral shank, and a subset of motion parameters of the unilateral shank (more detailed parameter definitions were described in Table S1). Figure 2Ci shows the results of the input parameter optimizations for the personal identification model. Figure 2D schematizes the structure of the LSTM neural network model. The model consisted of an LSTM layer and a classification layer (fully connected layer and softmax output). The number of neurons in the LSTM layer was optimized as 200. The time length of input data was optimized as 2 s (Table S3).

The limb motion dataset of 16 subjects at walking and running speeds of *v*_1_ (0.56, 0.83, 1.11, 1.39, 1.67, 1.94, 2.22, 2.5, and 2.78 m/s, respectively) in the first experiment was used to train the network model, and the limb motion dataset at different locomotion speeds of *v*_2_ (0.69, 0.97, 1.25, 1.53, 1.81, 2.08, 2.36, and 2.64 m/s, respectively) in the second experiment was used to test the trained model. The speeds in the training and test datasets were different to ensure feasibility across different speeds. It could be seen from Fig. 2Ci that the input parameter configuration (*v-w*) with motion velocity (*v*) and angular rate (*w*) of a single shank reached the highest accuracy compared to other parameter configurations. It was noted that the accuracy of both legs (*TwoLegs*) shows an unobvious advantage compared to that of a single shank (*v-w*). The configuration with the motion parameters of a single shank could simplify the wearable system and reduce the cost and the alignment complexity of the device. The personal identification result was shown in Fig. 2Ei, and it reached a high accuracy of 98.7% in recognizing 16 subjects.

Furthermore, we conducted the motion-state recognition for 6 subjects (subjects 1 to 6) during various motion states, including level walking and running, slope walking and slope running, and loaded walking, respectively. The limb motion dataset of 6 subjects at speeds of *v*_1_ in the first experiment was used to train the network model, and the limb motion dataset at different locomotion speeds of *v*_2_ in the second experiment was used to test the model. We also preoptimized the input parameters of the motion-state recognition model as shown in Fig. 2Cii and determined the best parameter configuration was the combination of the motion velocity and angular rate (i.e., *v-w*) of a single shank. Figure 2Eii indicated that an overall accuracy of 93.7% was reached in recognizing 5 different motion states for 6 subjects.

### Real-time estimation of locomotion speed

**Fig. 3.**
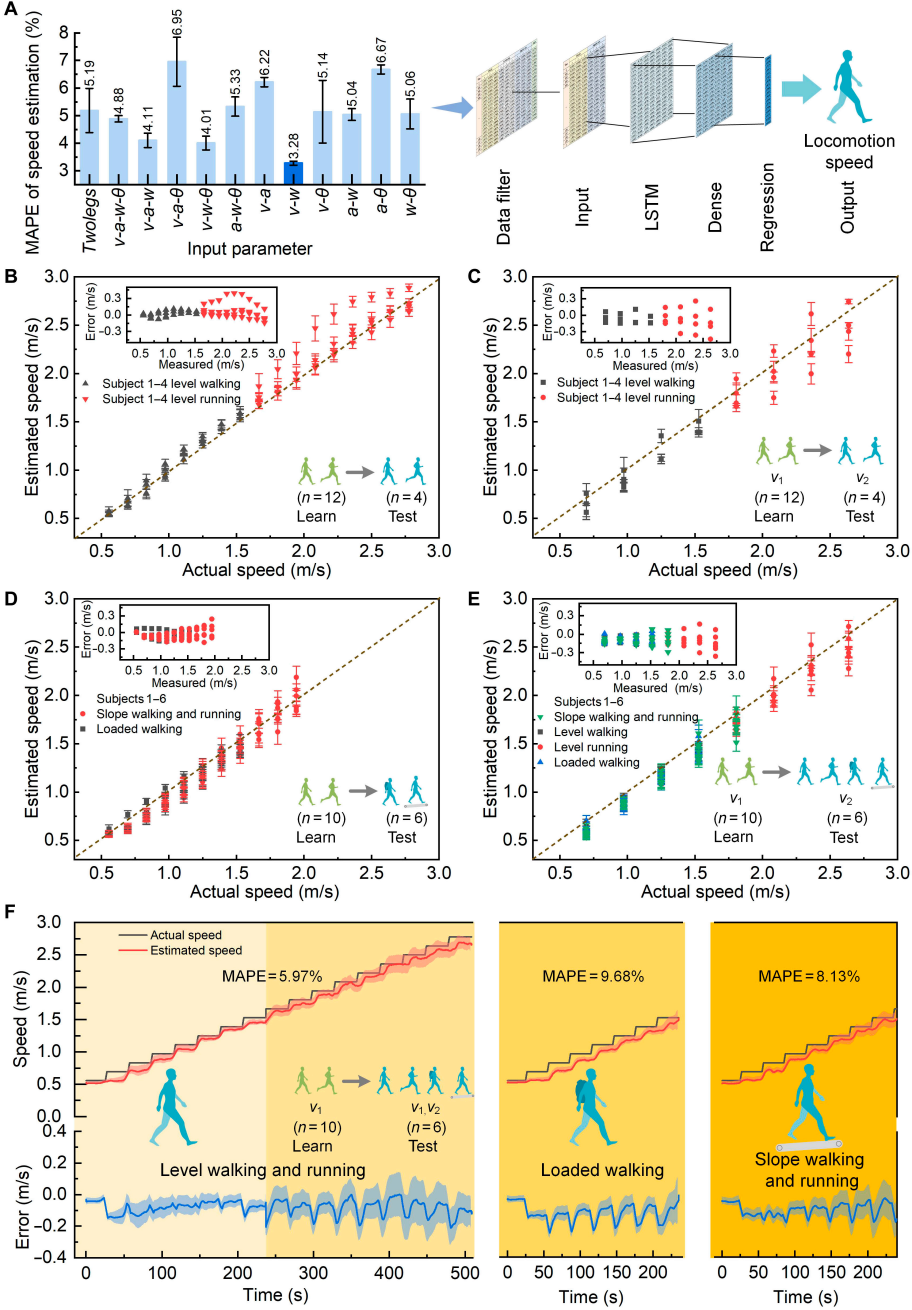
Locomotion speed estimation using the wearable system. (A) Results of speed estimation using different input parameter configurations and schematic diagram of LSTM-based neural network model as speed estimator. The best configuration with the minimum estimation error was highlighted in a darker blue. (B) Speed estimations for new subjects. The actual speed referred to the treadmill speed. A dataset of 4 new subjects was used to calculate the error bars. (C) Speed estimations for new subjects at new locomotion speeds. A dataset of 4 new subjects was used to calculate the error bars. (D) Speed estimations for new subjects across new motion states. A dataset of 6 new subjects was used to calculate the error bars. (E) Speed estimations for new subjects across new motion states at new locomotion speeds. A dataset of 6 new subjects was used to calculate the error bars. (F) Time-varying speed estimations for new subjects across new motion states. A dataset of 6 new subjects was used to calculate the error bars.

Although individual gaits showed significant differences as mentioned above, an interesting finding is that the kinetic-energy-related locomotion speed and metabolic rate could be extracted from the lower limb movements regardless of individual differences. To estimate the locomotion speed in real time using the wearable system, we built an LSTM neural network as shown in Fig. 3A. We optimized the input parameters of the speed estimator based on a criterion of minimizing the error of estimated locomotion speed. Figure 3A showed that the optimum input configuration with the motion velocity and angular rate (*v-w*) of one shank reached the minimum mean absolute percentage error (MAPE) of 3.28%. Therefore, we utilized the *v-w* parameters as the inputs of the speed estimator. In the above optimization experiment, the limb motion dataset of 16 subjects at the walking and running speeds of *v*_1_ in the first experiment was used to train the estimator, and the limb motion dataset at the different walking and running speed of *v*_2_ in the second experiment was used to test the estimator. To validate the generalization of the speed estimator for new subjects across new speeds and new motion states, we conducted the following experiments.

First, the motion dataset of 12 subjects during level walking and running in the first experiment was used to train the estimator, and the motion dataset of the other 4 new subjects in the second experiment was used to test the estimator. Figure 3B shows one example of the estimation results that the data-driven model trained from 12 subjects (subjects 5 to 16) was used to estimate 4 new subjects (subjects 1 to 4). The cross-validation results for other permutations of subjects are shown in Fig. S2. The results indicated that the level walking and running speed of new subjects could be estimated to reach an MAPE of 3.04 to 5.05% (3.96% on average) and a root mean square error of less than 0.4 m/s using the estimator without model recalibration. It showed good generalizability of the speed estimator for new subjects.

Second, the training dataset and test dataset were collected at different locomotion speeds to further validate the speed generalization of the estimator. Specifically, the motion dataset of 12 subjects (subjects 5 to 16) at walking and running speeds of *v*_1_ in the first experiment was used as the training dataset, and the motion dataset of 4 new subjects (subjects 1 to 4) at the locomotion speed of *v*_2_ in the second experiment was used to test the estimator. In this experiment, the MAPE of the estimated locomotion speed reached 5.06 to 6.74% (5.78% on average; Fig. 3C; the cross-validation results for other permutations of subjects were shown in Fig. S3).

Furthermore, we evaluated the generalizability of the speed estimator for new motion states. We used the motion dataset of 10 subjects (subjects 7 to 16) during level walking and running conditions in the first experiment as the training dataset and tested the trained estimator for 6 new subjects (subjects 1 to 6) during their loaded walking, slope walking, and slope running in the second experiment. The MAPE of the estimated locomotion speed for the new subjects across new motion states reached 5.18% (Fig. 3D).

Finally, we comprehensively evaluated the generalizability of the locomotion speed estimator across more complex conditions, for new subjects during new motion states at new motion speeds. The motion dataset of 10 subjects (subjects 7 to 16) during level walking and running at the speed of *v*_1_ in the first experiment was used as the training dataset, and the trained estimator was tested for 6 new subjects (subjects 1 to 6) across various conditions (level walking, level running, loaded walking, slope walking, and slope running) at a speed of *v*_2_ in the second experiment. The results were shown in Fig. 3E, and the MAPE was estimated to be 7.34%.

To meet the requirement of real-time monitoring during time-varying conditions, we further test the time-varying performance of the speed estimator. Figure 3F showed a continuously estimated locomotion speed using the trained estimator in Fig. 3E for 6 new subjects (subjects 1 to 6) during a time-varying level walking and running (0.56 to 2.78 m/s), a time-varying loaded walking (0.56 to 1.53 m/s), and a time-varying slope walking and running (0.56 to 1.94 m/s), respectively. It could be seen that the wearable system exhibited good real-time locomotion speed estimation for new subjects across various time-varying conditions and the maximum MAPE of the estimated locomotion speed was less than 10%.

### Real-time estimation of metabolic energy expenditure

**Fig. 4.**
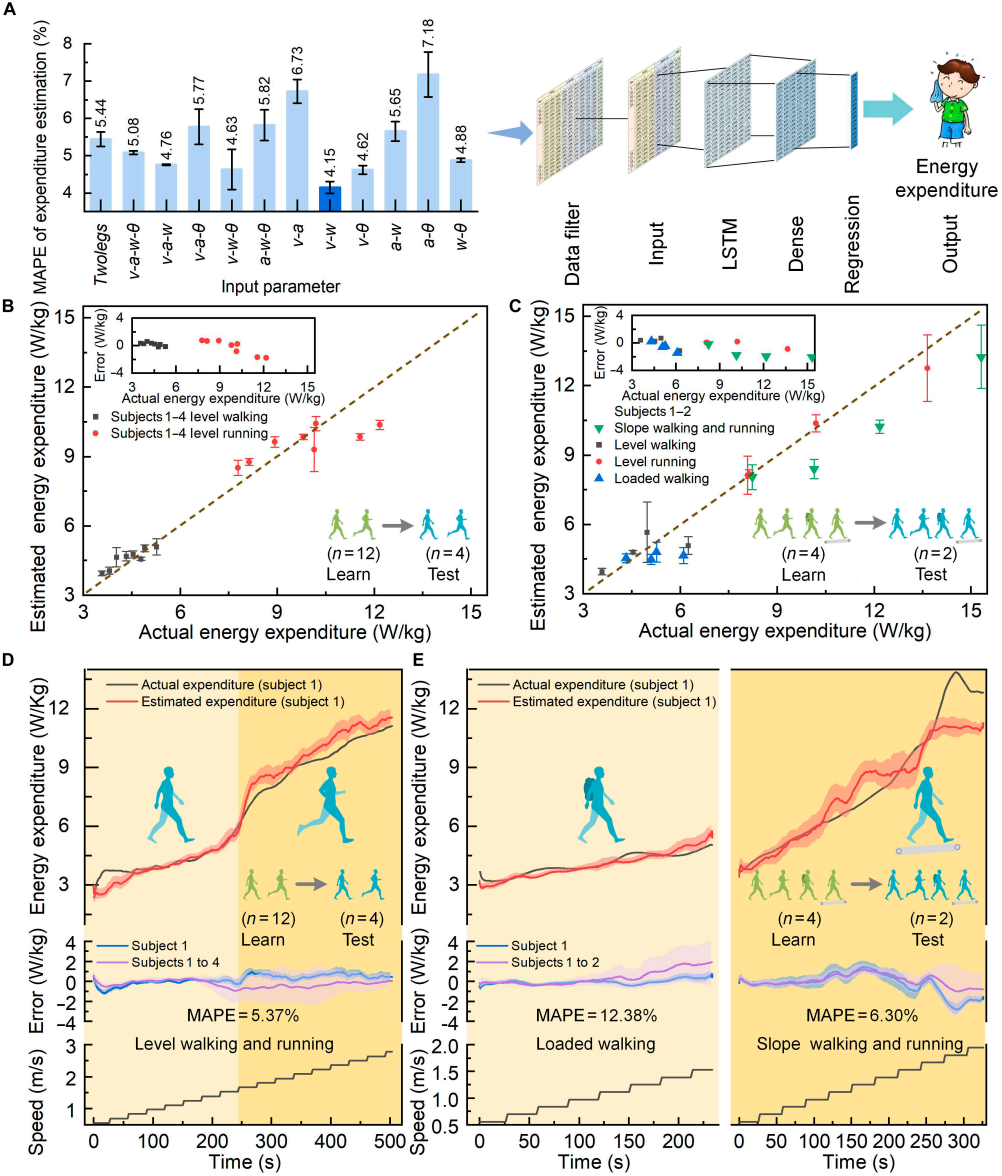
Metabolic energy expenditure estimation using the wearable system. (A) Results of metabolic energy estimation using different input parameters and schematic diagram of LSTM-based neural network model as energy estimator. The best configuration with the minimum estimation error was highlighted using a darker color. (B) Metabolic energy expenditure estimations for 4 new subjects during level walking and running. The actual energy expenditure refers to the ground-truth expenditure measured by the respiratory oxygen consumption meter. A dataset of 4 new subjects was used to calculate the error bars. (C) Metabolic energy expenditure estimation for 2 new subjects during various motion states (level walking and running, slope walking and running, and loaded walking). A dataset of 2 new subjects was used to calculate the error bars. (D) Time-varying metabolic energy expenditure estimations for new subjects during long-term time-varying walking and running. (E) Time-varying metabolic energy expenditure estimations for new subjects during loaded walking (left), slope walking, and running (right). In (D) and (E), the red and blue error bars were calculated from the dataset of subject 1 over the time. The purple error bars were calculated from the dataset of subjects 1 to 4 over the time.

Besides locomotion speed, metabolic rate is another key metric for assessing physical motor function. Daily energy expenditure during common activities of walking and running accounts for the majority of the body’s daily calorie consumption. Leg movements undertake major movements during walking and running. Therefore, the leg movement parameters are positively correlated with metabolic energy expenditure. However, an analytic relationship between the leg movement parameters and the metabolic energy expenditure is hard to be established because of complex leg dynamic behavior. Therefore, we propose a machine learning method to estimate the metabolic energy from the detected leg movement parameters in real time. To estimate the metabolic energy expenditure during walking and running, we built an LSTM neural network as shown in Fig. 4A. We optimized the input parameters of the metabolic energy estimator based on a criterion of minimizing the error of estimated metabolic expenditures. In the optimization experiment, the motion dataset of 16 subjects during the level walking and running at the speed of 1.25, 1.53, 1.94, and 2.50 m/s in the first experiment was used as the training dataset, and their motion dataset in the second experiment was used for the test. The ground-truth metabolic energy expenditure was measured synchronously using a spiroergometry system in the experiments. Figure 4A showed that the optimum parameter configuration of *v-w* reached the minimum MAPE of 4.15% for the metabolism estimator. Therefore, we utilized the *v-w* parameters as the inputs of the metabolism estimator hereinafter.

To validate the generalizability of the metabolism estimator for new subjects, we conducted the following experiments. The motion dataset of 12 subjects (subjects 5 to 16) during their level walking and running at the speed of 1.25, 1.53, 1.94, and 2.50 m/s in the first experiment was used as the training dataset, and the dataset of 4 new subjects (subjects 1 to 4) in the second experiment was used for the test. The test results were shown in Fig. 4B, and the cross-validation results for other permutations of subjects were shown in Fig. S4. The MAPE of the metabolic energy estimation for 4 new subjects during their level walking and running reached 6.23 to 7.73% (7.2% on average). Furthermore, more motion states including level walking and running, loaded walking, slope walking, and running were also involved. Figure 4C and Fig. S5 showed the estimation results for new subjects across various motion states, reaching an MAPE of 6.20 to 9.78% (8.45% on average), which was less than the error of state-of-the-art methods (e.g., 13%) [20].

It was noted that the above experiments were conducted under steady-state conditions. The walking or running activity during steady-state conditions was kept at each specific speed for 5 min. To avoid fatigue, each subject took a rest for at least 5 min between conditions. To test the time-varying performance of the energy expenditure estimator, 16 subjects performed long-term walking and running at time-varying speeds. Each subject increased his speed from 0.56 to 2.78 m/s with an ascending interval of 0.14 m/s, and each speed lasted 0.5 min. The whole period of the time-varying condition continued for 8.5 min. To validate the generalizability across individuals, the time-varying motion dataset of 12 subjects (subjects 5 to 16) in the first experiment was used as the training dataset, and the dataset of 4 new subjects (subjects 1 to 4) during the time-varying condition in the second experiment was used as the test dataset. Figure 4D shows the real-time estimated metabolic energy of the 4 new subjects during the time-varying level walking and running. Other cross-validation results were shown in Figs. S6 to S9. The MAPE of metabolic estimation using the wearable system during the time-varying conditions was 4.66 to 12.08% (7.57% on average).

Furthermore, to test metabolic estimates during various time-varying conditions, the dataset of 4 subjects (subjects 3 to 6) during time-varying loaded walking (0.56 to 1.53 m/s), slope walking, and running (0.56 to 1.94 m/s) was used as the training dataset, and the dataset of 2 new subjects (subjects 1 and 2) was used as the test dataset. Figure 4E showed the real-time estimated metabolic energy during the time-varying loaded walking, slope walking, and running for new subjects. Other cross-validation results were shown in Figs. S10 to S12. The wearable system achieved an MAPE of 4.18 to 14.71% (8.76% on average) in estimating real-time metabolic energy for new subjects across all time-varying conditions, significantly less than the error of state-of-the-art methods (e.g., 23%) [20]. The wearable system performed accurate estimates of time-varying metabolic energy expenditure and achieved good generalizability for new subjects.

### Multimodal assessments of human motion using the wearable leg movement monitoring system

**Fig. 5.**
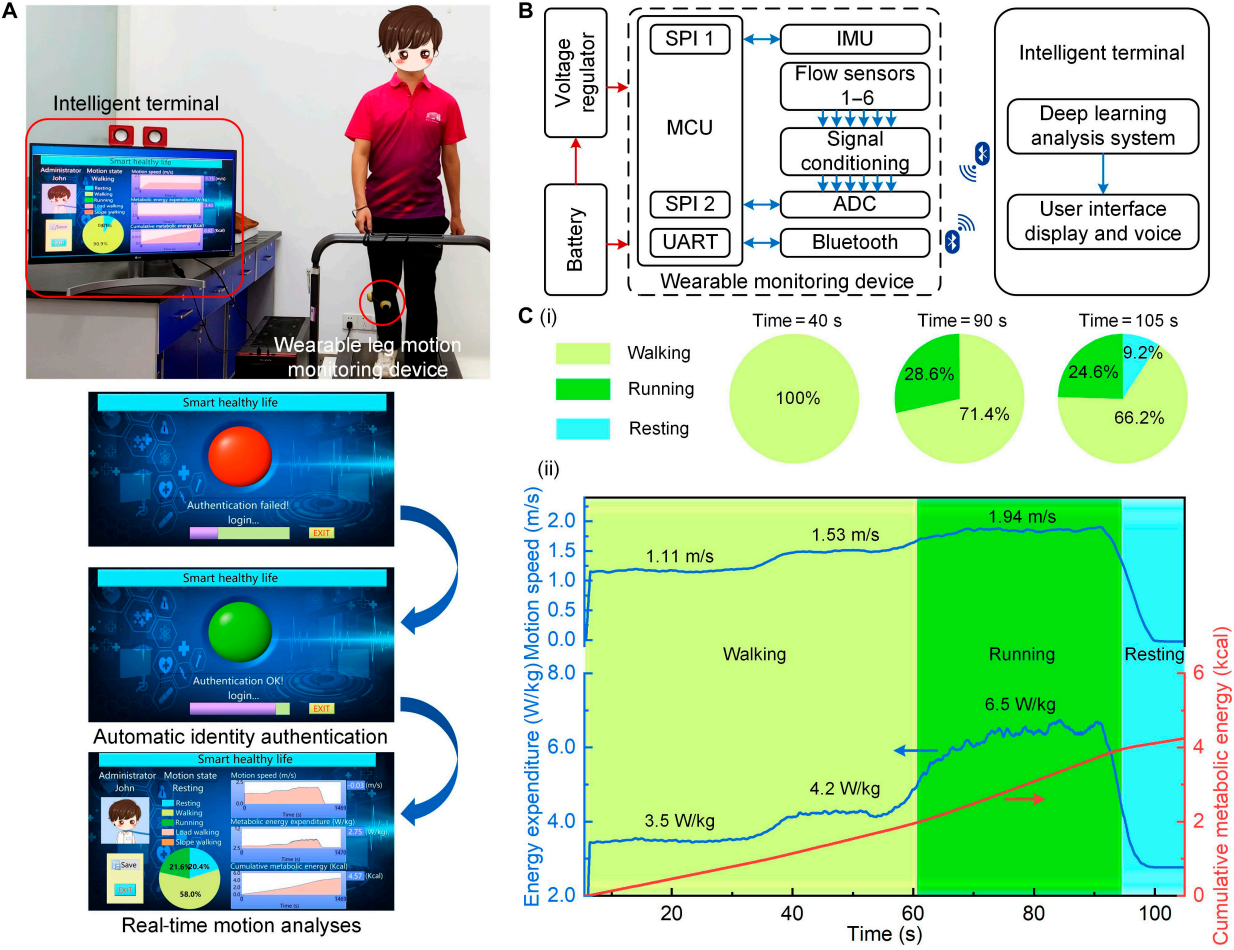
Demonstration of human motion analyses using the wearable system. (A) System setup and experimental scene. A subject walked/ran on a treadmill. A wearable leg motion monitoring device was worn on the shank and the intelligent terminal received the motion data through Bluetooth and performed the motion analyses in real time. The workflow involved automatic identity authentication and real-time motion analyses. (B) Hardware schematic diagram of the system. The wearable leg monitoring device comprised a homemade microflow sensor, an IMU, a signal conditioning circuit, an microcontrol unit, and a Bluetooth module. The intelligent terminal performed deep-learning-based analyses and displayed or reported the results. (C) The results of the multimodal analysis. (i) Proportion of various motion states during different times. (ii) Real-time monitoring of the locomotion speed, metabolic energy expenditure, and cumulative metabolic energy.

To showcase the functions of the proposed wearable system in our daily life, we built a system (Fig. 5A) consisting of our leg movement monitoring device worn on the shank of a subject and an intelligent terminal (a personal computer as an example) to implement automatic identity authentication, human motion analyses, and reports in real time. The shank motion of the subject was monitored by the wearable device, the motion data were transmitted wirelessly to the intelligent terminal, and the terminal automatically authenticated personal identity according to his/her gait in 2 s. After the user was authenticated successfully, the intelligent terminal started analyzing his/her leg motion data to recognize the motion state and estimate the walking/running speed and metabolic energy expenditure in real time. The hardware schematic diagram of the system setup was shown in Fig. 5B. The wearable system performed wireless communication with the intelligent terminal through Bluetooth, and the intelligent terminal carried out the online multimodal assessments of human motion and displayed the assessment results on a graphical user interface or reported the results by voice.

Figure 5C showed an example of the assessment results of the subject in the walking/running experiment. In the first 60 s, the subject walked at the speeds of 1.11 and 1.53 m/s consecutively and then speeded up to run at the speed of 1.94 m/s during 60 to 90 s, and, finally, the subject slowed down to stop during 90 to 105 s. Figure 5Ci showed the pie charts indicating the proportion of various motion states during different times. Figure 5Cii showed the real-time locomotion speed and metabolic energy expenditure during walking and running. The cumulative metabolic energy during the walking and running was calculated from the estimated metabolic rate (detail in Materials and Methods). The dynamic process of the above experiment was shown in Movie S1. The experiment demonstrated the wearable system was able to continuously monitor the leg movements and analyze the motion data in real time to recognize personal identity and motion states and accurately estimate motion energy during walking and running.

According to the results, the movement data of unilateral shank, without data from both legs, were sufficient to characterize the kinematics and kinetics of the whole body, which simplified the wearable system. The combination of the motion velocity and angular rate (*v-w*) of a single shank was proved to be the best input configuration of the fusion models for versatile perception tasks, including recognizing personal identity (achieving a recognition accuracy of 98.7% shown in Fig. 2Ci), identifying motion states (reaching an accuracy of 93.7% shown in Fig. 2Cii), estimating locomotion speed (reaching an error of 3.28% in Fig. 3A), and estimating metabolic energy expenditure (achieving an error of 4.15% shown in Fig. 4A). However, using accelerometer-detected acceleration gained lower accuracies shown in Figs. 2C, 3A, and 4A. The results concluded that the shank motion velocity was preferable to the accelerometer-detected acceleration in assessing human motor function. The reason was that the accelerometer detected the total acceleration of gravity and motion acceleration and was also suffered from shock and vibration interference during walking and running. To derive the velocity and attitude angle, the typical integral calculation of the accelerometer-detected acceleration would result in significant errors. In this work, we utilized a microflow sensor to detect a motion-induced surface flow for measuring limb motion velocity. The measured motion velocity could accurately estimate the motion acceleration, being resilient to gravity and shock interference. Therefore, the shank angle could be estimated without the necessity of integral calculations and thus achieved drift-free measurements.

The developed leg movement monitoring system was competent in human motor function assessment in daily life. People could monitor their daily activities, comprehensively understand their exercise energy expenditures, and make motor management of daily activity for preventing obesity or cardiovascular disease and rehabilitation training. With the real-time monitoring of leg movement, people could make daily living seamless and convenient, such as safe and easy access to living facilities by personal gait ID and easy interaction with smart devices. It would be also useful and securer for organizations or companies to adopt personal gaits into their identity authentication systems.

This work would also provide effective approaches for researchers in other fields. Medical researchers could use the wearable system to study the correlation between motion indicators and health problems and provide health guidance on physical activity. Engineers might consider incorporating velocity sensors into the configuration of human motion monitoring systems to acquire more accurate and reliable measurements. Researchers in ergonomics and biomechanics could study the gait patterns for detecting abnormal gaits and guiding athletic competition and daily training. In physiotherapy, accurate gait analysis could guide rehabilitation programs to improve outcomes.

## Conclusion

We proposed a new wearable leg movement monitoring system for accurately detecting leg movements and quantitively assessing motions during human walking and running. The shank movement parameters were fused to implement real-time multimodal perceptions on personal identification, motion state recognition, and estimates of locomotion speed and metabolic energy during steady-state and time-varying motion conditions. We proposed an optimum configuration of the sensing parameters to maximize the accuracies of feature recognition and energy estimation while simplifying the wearable system. The experimental result showed that the combination of motion velocity and angular rate of one shank achieved the most accurate performances for versatile tasks. It also validated the reliability and superiority of the wearable system in terms of multimodality, accuracy, and long-term stability. In future work, we plan to perform more experiments on more subjects across more human activities to further improve the generalization and robustness of the system. We also plan to extend the method in the future to diagnose motor function injury and implement exoskeleton control.

## Materials and Methods

### Motion parameters and subject experiments on the treadmill

Sixteen healthy subjects (subjects 1 to 16) participated in the motion experiments. They (*n* = 16 men; age, 25.2 ± 2.9 years; height, 1.74 ± 0.05 m; body mass, 67.3 ± 7.4 kg) walked or ran on a treadmill (R1, King Smith, China). The locomotion speeds were set by the treadmill. The motion states in the experiments were described as follows:

• Level walking: All subjects experienced level walking at a steady-state speed of 1.25 and 1.53 m/s, respectively, each speed lasting for 5 min. All subjects experienced a time-varying walking at a step-by-step ascending speed (0.56, 0.69, 0.83, 0.97, 1.11, 1.25, 1.39, and 1.53 m/s successively, each speed lasted 0.5 min).
• Level running: All subjects experienced level running at a steady-state speed of 1.94 and 2.50 m/s, respectively, each speed lasted for 5 min. All subjects experienced a time-varying running at a step-by-step ascending speed (1.67, 1.81, 1.94, 2.08, 2.22, 2.36, 2.50, 2.64, and 2.78 m/s successively, each speed lasted 0.5 min).
• Loaded walking: 6 subjects (subjects 1 to 6) experienced loaded walking. During the loaded walking, the subject carried a 12-kg backpack. The walking speed was set the same as that during the level walking state.
• Slope walking: 6 subjects (subjects 1 to 6) experienced slope walking on a sloped treadmill. The angle of the slope was 7°. The walking speed was set the same as that during the level walking state.
• Slope running: 6 subjects (subjects 1 to 6) experienced slope running at a speed of 1.94 m/s on a sloped treadmill for 5 min. The angle of the slope was 7°. Six subjects experienced the time-varying running at a step-by-step ascending speed (1.67, 1.81, and 1.94 m/s successively, each speed lasting 0.5 min).

All motion states were tested twice. The subject removed and rewore the monitoring device between 2 experiments to validate the feasibility and the generalization across different wearing. One experiment was used for training the network model, and another experiment was used for testing the trained model. Between the 2 experiments, the subjects removed and rewore the devices, resulting in the wearing difference for evaluating the robustness of the trained network model across different wearing. The detailed experimental conditions were described in Table S2. During the above experiments, all subjects wore a spiroergometry system (METALYZER 3B, CORTEX Biophysik Co. Ltd.) to measure their metabolic energy expenditure synchronously.

### Data acquisition and data sample processing

The motion velocity, acceleration, angular rate, and angle of one shank were detected using our custom-made wearable devices at a sampling frequency of 100 Hz. For personal identification and motion-state recognition, the time length of input data was optimized as 2 s. The 2-s window of data was shifted and updated in real time. For the real-time motion speed estimation and the real-time metabolic energy expenditure estimation, the time length of the input data was optimized as 4 s. The 4-s window of data was shifted and updated in real time. The detailed optimization processes were described in Fig. S13 and Table S3.

### Hardware and software design of the wearable device

The wearable device weighed 69.1 g and had a power consumption of about 450 mW. A low-power Bluetooth module (DA14580, Dialog Semiconductor) was used for wireless data transmission. A rechargeable lithium battery was used for the power supply. The device could work continuously for more than 3.5 h using a 600-mAh lithium battery. Data processing, calculation, and storage were performed on the MATLAB R2022a platform. The intelligent terminal software was designed using NI LabVIEW2018.

### Accuracy calculation of personal identification and motion state recognition

For personal identification, the accuracy of one subject (e.g., subject 1) can be calculated as follows:

$${\text{Accuracy}}_{\text{Subject}\;1}=\frac{n_{1}}{N_{1}}\times 100\%$$ (1)

where *N*_1_ refers to the number of test samples for subject 1 and *n*_1_ refers to the number of samples correctly identified as subject 1. Similarly, the accuracy of subjects 2 to 16 can be calculated.

The overall accuracy of personal identification can be calculated as follows:

$${\text{Accuracy}}_{\text{Overall}}=\frac{n_{1}+n_{2}+n_{3}+\cdots +n_{16}}{N_{1}+N_{2}+N_{3}+\cdots +N_{16}}\times 100\%$$ (2)

where *N*_2_, *N*_3_, …, *N*_16_ refer to the number of test samples for subjects 2 to 16 and *n*_2_, *n*_3_, …, *n*_16_ refer to the number of samples correctly identified as subjects 2 to 16.

Similarly, the accuracy of motion-state recognition can also be calculated.

### Calculation of the cumulative metabolic energy

The cumulative metabolic energy (in kilocalories) was denoted as *E*_c_ and calculated by:

$$E_{c}=0.0002389\times \int E_{p}dt\cdot W$$ (3)

where *E*_p_ was the estimated metabolic rate (in watts per kilogram) and *W* was the weight of the subject.

Experiments performed in this study involving human participants were approved by the Institution Review Board of Tsinghua University (no. 20180009). In addition, informed consent was obtained from human subjects to use their images and conduct the experiments described in this paper.

## Data Availability

The data are available from the corresponding author on reasonable request.

## Supplementary Materials

Supplementary 1 Movie S1

Supplementary 2 Tables S1 to S3 Note S1 Figs. S1 to S13
