# Supplementary material for: Wearable Leg Movement Monitoring System for High-Precision Real-Time Metabolic Energy Estimation and Motion Recognition
Source: Research (Wash D C). 2023 Aug 23;6:0214. doi: 10.34133/research.0214 (PMC11918258; doi:10.34133/research.0214)
Supplement: Supplementary 2 — Tables S1 to S3 Note S1 Figs. S1 to S13 [file research.0214.f2.docx]

Supplementary Materials

**Wearable leg movement monitoring system for high-precision real-time metabolic energy estimation and motion recognition**

*Jinfeng Yuan, Yuzhong Zhang, Shiqiang Liu* and Rong Zhu**

*Corresponding author. Email: zr_gloria@mail.tsinghua.edu.cn, liushiqiang@mail.tsinghua.edu.cn

**This PDF file includes:**

Tables S1 to S3

Note S1

Figure S1 to S13

**Table S1.** Label interpretation of the input parameters

| Input parameters | Full name of the parameter | | | | |
| --- | --- | --- | --- | --- | --- |
|  | Velocity | Acceleration | Angular rate | Angle | Remarks |
| *TwoLegs* | ✓ | ✓ | ✓ | ✓ | Right and left shank data |
| *v-a-w-θ* | ✓ | ✓ | ✓ | ✓ | Single shank data |
| *v-a-w* | ✓ | ✓ | ✓ |  |  |
| *v-a-θ* | ✓ | ✓ |  | ✓ |  |
| *v-w-θ* | ✓ |  | ✓ | ✓ |  |
| *a-w-θ* |  | ✓ | ✓ | ✓ |  |
| *v-a* | ✓ | ✓ |  |  |  |
| *v-w* | ✓ |  | ✓ |  |  |
| *v-θ* | ✓ |  |  | ✓ |  |
| *a-w* |  | ✓ | ✓ |  |  |
| *a-θ* |  | ✓ |  | ✓ |  |
| *w-θ* |  |  | ✓ | ✓ |  |

“TwoLegs” refers to the all motion parameters of two shanks, including velocity(*v*), acceleration(*a*), angular rate(*w*) and angle(*θ*); the remaining label refer to the corresponding motion parameters of a single shank, we used the parameters of the right shank in this work.

**Table S2.** Specific experimental conditions for each subject

|  | Level walking and running^*^ | Level walking^**^ | | Level running^**^ | | Loaded walking^*^ | Loaded walking^**^ | | Slope walking and running^*^ | Slope walking^**^ | | Slope running^**^ |
| --- | --- | --- | --- | --- | --- | --- | --- | --- | --- | --- | --- | --- |
|  |  |  | |  | |  | | |  | | | |
|  | Speed (m/s) | | | | | | | | | | | |
|  | 0.56-2.78 | 1.25 | 1.53 | 1.94 | 2.5 | 0.56-1.53 | 1.25 | 1.53 | 0.56-1.94 | 1.25 | 1.53 | 1.94 |
| Subject 1 | ✓ | ✓ | ✓ | ✓ | ✓ | ✓ | ✓ | ✓ | ✓ | ✓ | ✓ | ✓ |
| Subject 2 | ✓ | ✓ | ✓ | ✓ | ✓ | ✓ | ✓ | ✓ | ✓ | ✓ | ✓ | ✓ |
| Subject 3 | ✓ | ✓ | ✓ | ✓ | ✓ | ✓ | ✓ | ✓ | ✓ | ✓ | ✓ | ✓ |
| Subject 4 | ✓ | ✓ | ✓ | ✓ | ✓ | ✓ | ✓ | ✓ | ✓ | ✓ | ✓ | ✓ |
| Subject 5 | ✓ | ✓ | ✓ | ✓ | ✓ | ✓ | ✓ | ✓ | ✓ | ✓ | ✓ | ✓ |
| Subject 6 | ✓ | ✓ | ✓ | ✓ | ✓ | ✓ | ✓ | ✓ | ✓ | ✓ | ✓ | ✓ |
| Subject 7 | ✓ | ✓ | ✓ | ✓ | ✓ | NA | | | | | | |
| Subject 8 | ✓ | ✓ | ✓ | ✓ | ✓ |  |  |  |  |  |  |  |
| Subject 9 | ✓ | ✓ | ✓ | ✓ | ✓ |  |  |  |  |  |  |  |
| Subject 10 | ✓ | ✓ | ✓ | ✓ | ✓ |  |  |  |  |  |  |  |
| Subject 11 | ✓ | ✓ | ✓ | ✓ | ✓ |  |  |  |  |  |  |  |
| Subject 12 | ✓ | ✓ | ✓ | ✓ | ✓ |  |  |  |  |  |  |  |
| Subject 13 | ✓ | ✓ | ✓ | ✓ | ✓ |  |  |  |  |  |  |  |
| Subject 14 | ✓ | ✓ | ✓ | ✓ | ✓ |  |  |  |  |  |  |  |
| Subject 15 | ✓ | ✓ | ✓ | ✓ | ✓ |  |  |  |  |  |  |  |
| Subject 16 | ✓ | ✓ | ✓ | ✓ | ✓ |  |  |  |  |  |  |  |

* The subjects experienced locomotion with a step-by-step ascending speed (0.56 m/s, 0.69 m/s, 0.83 m/s, 0.97 m/s, 1.11 m/s, 1.25 m/s, 1.39 m/s, 1.53 m/s, 1.67 m/s, 1.81 m/s, 1.94 m/s, 2.08 m/s, 2.22 m/s, 2.36 m/s, 2.50 m/s, 2.64 m/s, 2.78 m/s) and maintain 0.5 minutes for each speed.

** The subjects experienced locomotion with constant speed (1.25 m/s, 1.53 m/s, 1.94 m/s, 2.50 m/s) and maintain 5 minutes for each speed. Subjects wore a respiratory oxygen consumption meter to evaluate the metabolic energy expenditure for 5 minutes at the same time.

**Note S1.** We propose a data fusion method to figure out tri-axis motion velocity in real-time based on a long short-term memory (LSTM) neural network. The signal outputs of two flow sensors are used as the inputs and the tri-axis motion velocities are used as the outputs. The training datasets are collected from a training experiment when the subject walks and runs on a treadmill with a velocity increasing from 0 to 2.78 m/s at an interval of 0.28 m/s, and each speed lasts for 30 s. The testing datasets are collected from a testing experiment when the subject walks and runs on a treadmill at a step-by-step descending speed (2.78 m/s, 2.22 m/s, 1.67 m/s, 1.39 m/s, 1.11 m/s, 0.83 m/s, 0.56 m/s), and each speed lasts for 50 s. The sampling rate of the sensor signal is 100 Hz. The time window length of the training and testing data sample is optimized as 0.4 s. The neural network model consists of a LSTM hidden layer and a fully connected layer. The number of neurons in the LSTM layer is 200. The root mean square error (RMSE) is used as a loss function. The model training is carried out in MATLAB software. True values of tri-axis motion velocity are captured by using an optical VICON system. The testing results are shown in Figure S1. It can be seen that the micro velocity sensor can accurately measure the dynamic tri-axis motion velocity in real-time. The RMSE reaches 0.054 m/s.

**Figure S1. Real-time tri-axis motion velocity measured by our wearable device with the data fusion method.** a) Comparison and error between estimated and true values of X-axis motion velocity. b) Partially enlarged details of Figure S1a. c) Comparison and error between estimated and true values of Y-axis motion velocity. d) Partially enlarged details of Figure S1c. e) Comparison and error between estimated and true values of Z-axis motion velocity. f) Partially enlarged details of Figure S1e.

**Figure S2. Cross-validation results of locomotion speed estimation for new subjects.** Samples of 4 subjects are used to calculate the error bar. **a)** The datasets of 12 subjects (Subject 5 to 16) were used to train the estimator, and the datasets of new untrained 4 subjects (Subject 1 to 4) were used to test the estimator. **b)** The datasets of 12 subjects (Subject 1 to 4, Subject 9 to 16) were used to train the estimator, and the datasets of new untrained 4 subjects (Subject 5 to 8) were used to test the estimator. **c)** The datasets of 12 subjects (Subject 1 to 8, Subject 13 to 16) were used to train the estimator, and the datasets of new untrained 4 subjects (Subject 9 to 12) were used to test the estimator. **d)** The datasets of 12 subjects (Subject 1 to 12) were used to train the estimator, and the datasets of new untrained 4 subjects (Subject 13 to 16) were used to test the estimator.

**Figure S3. Cross-validation results of locomotion speed estimation for new subjects and new locomotion speed.** Samples of 4 subjects are used to calculate the error bar. The speed *v*_1_ (0.56, 0.83, 1.11, 1.39, 1.67, 1.94, 2.22, 2.5, 2.78 m/s) were used as the training dataset, and the speed *v*_2_ (0.69, 0.97, 1.25, 1.53, 1.81, 2.08, 2.36, 2.64 m/s) were used as the test dataset. **a)** The datasets of 12 subjects (Subject 5 to 16) were used to train the estimator, and the datasets of new untrained 4 subjects (Subject 1 to 4) were used to test the estimator. **b)** The datasets of 12 subjects (Subject 1 to 4, Subject 9 to 16) were used to train the estimator, and the datasets of new untrained 4 subjects (Subject 5 to 8) were used to test the estimator. **c)** The datasets of 12 subjects (Subject 1 to 8, Subject 13 to 16) were used to train the estimator, and the datasets of new untrained 4 subjects (Subject 9 to 12) were used to test the estimator. **d)** The datasets of 12 subjects (Subject 1 to 12) were used to train the estimator, and the datasets of new untrained 4 subjects (Subject 13 to 16) were used to test the estimator.

**Figure S4. Cross-validation results of metabolic energy expenditure estimation for new subjects.** Samples of 4 subjects are used to calculate the error bar. **a)** The datasets of 12 subjects (Subject 5 to 16) were used to train the estimator, and the datasets of new untrained 4 subjects (Subject 1 to 4) were used to test the estimator. **b)** The datasets of 12 subjects (Subject 1 to 4, Subject 9 to 16) were used to train the estimator, and the datasets of new untrained 4 subjects (Subject 5 to 8) were used to test the estimator. **c)** The datasets of 12 subjects (Subject 1 to 8, Subject 13 to 16) were used to train the estimator, and the datasets of new untrained 4 subjects (Subject 9 to 12) were used to test the estimator. **d)** The datasets of 12 subjects (Subject 1 to 12) were used to train the estimator, and the datasets of new untrained 4 subjects (Subject 13 to 16) were used to test the estimator.

**Figure S5. Cross-validation results of metabolic energy expenditure estimation for new subjects during various motion states.** Samples of 2 subjects are used to calculate the error bar. **a)** The datasets of 4 subjects (Subjects 3 to 6) were used to train the estimator, and the datasets of new untrained 2 subjects (Subjects 1 to 2) were used to test the estimator. **b)** The datasets of 4 subjects (Subject 1 to 2, Subject 5 to 6) were used to train the estimator, and the datasets of new untrained 2 subjects (Subject 3 to 4) were used to test the estimator. **c)** The datasets of 4 subjects (Subjects 1 to 4) were used to train the estimator, and the datasets of new untrained 2 subjects (Subjects 5 to 6) were used to test the estimator.

**Figure S6. Cross-validation results of time-varying metabolic energy expenditure estimation for new subjects (Subjects 1 to 4).** The datasets of 12 subjects (Subjects 5 to 16) were used to train the estimator, and the datasets of new untrained 4 subjects (Subjects 1 to 4) were used to test the estimator. The error represents the error between the estimated energy expenditure and the actual energy expenditure measured by the respiratory oxygen consumption meter. The red and blue error bar is calculated from the estimation error of the corresponding subject in the last 10 seconds. The purple error bar is calculated from the estimation error of subjects 1 to 4 over the time. **a)** Time-varying metabolic energy expenditure estimation for subject 1. **b)** Time-varying metabolic energy expenditure estimation for subject 2. **c)** Time-varying metabolic energy expenditure estimation for subject 3. **d)** Time-varying metabolic energy expenditure estimation for subject 4.

**Figure S7. Cross-validation results of time-varying metabolic energy expenditure estimation for new subjects (Subjects 5 to 8).** The datasets of 12 subjects (Subjects 1 to 4, Subjects 9 to 16) were used to train the estimator, and the datasets of new untrained 4 subjects (Subjects 5 to 8) were used to test the estimator. The error represents the error between the estimated energy expenditure and the actual energy expenditure measured by the respiratory oxygen consumption meter. The red and blue error bar is calculated from the estimation error of the corresponding subject in the last 10 seconds. The purple error bar is calculated from the estimation error of subjects 5 to 8 over the time. **a)** Time-varying metabolic energy expenditure estimation for subject 5. **b)** Time-varying metabolic energy expenditure estimation for subject 6. **c)** Time-varying metabolic energy expenditure estimation for subject 7. **d)** Time-varying metabolic energy expenditure estimation for subject 8.

**Figure S8. Cross-validation results of time-varying metabolic energy expenditure estimation for new subjects (Subjects 9 to 12).** The datasets of 12 subjects (Subjects 1 to 8, Subjects 13 to 16) were used to train the estimator, and the datasets of new untrained 4 subjects (Subjects 9 to 12) were used to test the estimator. The error represents the error between the estimated energy expenditure and the actual energy expenditure measured by the respiratory oxygen consumption meter. The red and blue error bar is calculated from the estimation error of the corresponding subject in the last 10 seconds. The purple error bar is calculated from the estimation error of subjects 9 to 12 over the time. **a)** Time-varying metabolic energy expenditure estimation for subject 9. **b)** Time-varying metabolic energy expenditure estimation for subject 10. **c)** Time-varying metabolic energy expenditure estimation for subject 11. **d)** Time-varying metabolic energy expenditure estimation for subject 12.

**Figure S9. Cross-validation results of time-varying metabolic energy expenditure estimation for new subjects (Subjects 13 to 16).** The datasets of 12 subjects (Subjects 1 to 12) were used to train the estimator, and the datasets of new untrained 4 subjects (Subjects 13 to 16) were used to test the estimator. The error represents the error between the estimated energy expenditure and the actual energy expenditure measured by the respiratory oxygen consumption meter. The red and blue error bar is calculated from the estimation error of the corresponding subject in the last 10 seconds. The purple error bar is calculated from the estimation error of subjects 13 to 16 over the time. **a)** Time-varying metabolic energy expenditure estimation for subject 13. **b)** Time-varying metabolic energy expenditure estimation for subject 14. **c)** Time-varying metabolic energy expenditure estimation for subject 15. **d)** Time-varying metabolic energy expenditure estimation for subject 16.

**Figure S10. Cross-validation results of time-varying metabolic energy expenditure estimation for new subjects during loaded walking, slope walking and running (Subjects 1 to 2).** The datasets of 4 subjects (Subjects 3 to 6) were used to train the estimator, and the datasets of new untrained 2 subjects (Subjects 1 to 2) were used to test the estimator. The error represents the error between the estimated energy expenditure and the actual energy expenditure measured by the respiratory oxygen consumption meter. The red and blue error bar is calculated from the estimation error of the corresponding subject in the last 10 seconds. The purple error bar is calculated from the estimation error of subjects 1 to 2 over the time. **a)** Time-varying metabolic energy expenditure estimation for subject 1. **b)** Time-varying metabolic energy expenditure estimation for subject 2.

**Figure S11. Cross-validation results of time-varying metabolic energy expenditure estimation for new subjects during loaded walking, slope walking and running (Subjects 3 to 4).** The datasets of 4 subjects (Subjects 1, 2, 5 and 6) were used to train the estimator, and the datasets of new untrained 2 subjects (Subjects 3 to 4) were used to test the estimator. The error represents the error between the estimated energy expenditure and the actual energy expenditure measured by the respiratory oxygen consumption meter. The red and blue error bar is calculated from the estimation error of the corresponding subject in the last 10 seconds. The purple error bar is calculated from the estimation error of subjects 3 to 4 over the time. **a)** Time-varying metabolic energy expenditure estimation for subject 3. **b)** Time-varying metabolic energy expenditure estimation for subject 4.

**Figure S12. Cross-validation results of time-varying metabolic energy expenditure estimation for new subjects during loaded walking, slope walking and running (Subjects 5 to 6).** The datasets of 4 subjects (Subjects 1 to 4) were used to train the estimator, and the datasets of new untrained 2 subjects (Subjects 5 to 6) were used to test the estimator. The error represents the error between the estimated energy expenditure and the actual energy expenditure measured by the respiratory oxygen consumption meter. The red and blue error bar is calculated from the estimation error of the corresponding subject in the last 10 seconds. The purple error bar is calculated from the estimation error of subjects 5 to 6 over the time. **a)** Time-varying metabolic energy expenditure estimation for subject 5. **b)** Time-varying metabolic energy expenditure estimation for subject 6.

**Figure S13. Training and testing dataset establishment.** We use the methods of window interception and window shift to establish the model dataset. For the personnel identification and motion-state recognition, the time length of each sample is 2 s, and the time interval between samples (window shift time) is 0.5s. For the motion velocity and the metabolic energy expenditure estimation, the time length of each sample is 4 s, and the time interval between samples (window shift time) is 0.01 s. The optimal length of time and the optimal time interval can be seen in Table S3. The number of neurons in the LSTM layer of the deep learning system is 200.

**Table S3. The optimization processes of the time length and time interval of dataset establishment.** To simplify the optimization process, we selected the input parameters as “*v-a-w-θ*”. The motion datasets of 16 subjects during the first experiment were used as the training datasets, and their all motion datasets during the second experiment were used for the test. The optimal result is highlighted.

**a** The accuracy (%) of different parameters for personnel identification

| Time interval between sample (s) | Time length of the sample (s) | | | | |
| --- | --- | --- | --- | --- | --- |
|  | 0.5 | 1.0 | 2.0 | 4.0 | 6.0 |
| 0.5 | 91.93 | 92.94 | **95.89** | 95.66 | 95.02 |
| 1.0 | 90.01 | 91.56 | 91.76 | 95.49 | 93.11 |
| 2.0 | 83.42 | 82.36 | 87.92 | 91.13 | 90.20 |
| 4.0 | 72.07 | 75.68 | 79.04 | 89.60 | 72.86 |

**b** The accuracy (%) of different parameters for motion-state recognition

| Time interval between sample (s) | Time length of the sample (s) | | | | |
| --- | --- | --- | --- | --- | --- |
|  | 0.5 | 1.0 | 2.0 | 4.0 | 6.0 |
| 0.5 | 83.73 | 89.31 | **91.40** | 89.09 | 89.50 |
| 1.0 | 84.56 | 87.56 | 90.23 | 87.05 | 90.46 |
| 2.0 | 79.83 | 87.60 | 87.84 | 90.08 | 88.86 |
| 4.0 | 74.97 | 85.45 | 84.96 | 85.83 | 83.64 |

According to the optimization results of personnel identification and motion-state recognition, we can conclude that the time interval shall be as short as possible due to the enlargement of training samples is beneficial to improve the accuracy. Therefore, we set the time interval of estimation of locomotion velocity and metabolic energy expenditure as 0.01 s, and optimize the time length of the sample.

**c** The MAPE (%) of different parameters for estimation of locomotion velocity

| Time interval between sample (s) | Time length of the sample (s) | | | | |
| --- | --- | --- | --- | --- | --- |
|  | 2.0 | 3.0 | 4.0 | 5.0 | 6.0 |
| 0.01 | 4.61 | 4.80 | **4.39** | 4.68 | 5.08 |

**d** The MAPE (%) of different parameters for estimation of metabolic energy expenditure

| Time interval between sample (s) | Time length of the sample (s) | | | | |
| --- | --- | --- | --- | --- | --- |
|  | 2.0 | 3.0 | 4.0 | 5.0 | 6.0 |
| 0.01 | 5.55 | 4.79 | **4.63** | 4.89 | 5.85 |
